# Improved object detection method for autonomous driving based on DETR

**DOI:** 10.3389/fnbot.2024.1484276

**Published:** 2025-01-20

**Authors:** Huaqi Zhao, Songnan Zhang, Xiang Peng, Zhengguang Lu, Guojing Li

**Affiliations:** ^1^The Heilongjiang Provincial Key Laboratory of Autonomous Intelligence and Information Processing, School of Information and Electronic Technology, Jiamusi University, Jiamusi, China; ^2^School of Materials Science and Engineering, Jiamusi University, Jiamusi, China

**Keywords:** object detection, feature extraction, transformer encoder, loss function, parameter tuning

## Abstract

Object detection is a critical component in the development of autonomous driving technology and has demonstrated significant growth potential. To address the limitations of current techniques, this paper presents an improved object detection method for autonomous driving based on a detection transformer (DETR). First, we introduce a multi-scale feature and location information extraction method, which solves the inadequacy of the model for multi-scale object localization and detection. In addition, we developed a transformer encoder based on the group axial attention mechanism. This allows for efficient attention range control in the horizontal and vertical directions while reducing computation, ultimately enhancing the inference speed. Furthermore, we propose a novel dynamic hyperparameter tuning training method based on Pareto efficiency, which coordinates the training state of the loss functions through dynamic weights, overcoming issues associated with manually setting fixed weights and enhancing model convergence speed and accuracy. Experimental results demonstrate that the proposed method surpasses others, with improvements of 3.3%, 4.5%, and 3% in average precision on the COCO, PASCAL VOC, and KITTI datasets, respectively, and an 84% increase in FPS.

## 1 Introduction

Autonomous driving technology utilizes a combination of sensor technology, artificial intelligence, big data analysis and processing, and computer vision to enable computers to safely drive vehicles with partial or unmanned intervention. Object detection plays a crucial role in recognizing targets during autonomous driving and assists the central control system in providing necessary driving commands.

In autonomous driving scenarios, objects such as vehicles, pedestrians, and traffic signs are distributed across multiple scales depending on their distance. Distant pedestrians and traffic signs often appear as small targets, while nearby vehicles dominate the frame as large targets. Object size and appearance vary significantly due to differences in distance and angle. Multi-scale feature extraction addresses this challenge by capturing multi-level features simultaneously, enhancing the robustness of detecting objects across various scales (Lin et al., 2017). Additionally, complex backgrounds such as buildings and trees often interfere with object detection, while the dynamic nature of targets' such as moving pedestrians and vehicles' further complicates the task. Transformer-based architectures excel in modeling global contextual relationships, enabling them to adapt effectively to dynamic target variations, particularly in scenarios involving occlusions. Attention mechanisms offer a practical solution for resource-constrained embedded devices by efficiently allocating computational resources. By focusing computational power on key object regions, attention mechanisms significantly enhance real-time performance. Moreover, autonomous driving requires addressing multiple optimization objectives, such as the simultaneous classification and localization of objects. Adjusting and designing loss function weights can further optimize the model's multi-objective learning capabilities (Ou et al., 2023).

The integration of these technologies—multi-scale feature extraction, attention mechanisms, and optimized loss functions—provides greater accuracy, robustness, and efficiency for object detection in autonomous driving. These advancements establish a foundation for developing intelligent autonomous driving systems capable of reliable performance in complex real-world scenarios.

Object detection involves both traditional and deep-learning-based methods. Traditional methods typically include generating candidate frames, extracting features, and performing classification (Dalal and Triggs, 2005). Non-maximum suppression (NMS) (Neubeck and Van Gool, 2006) is then used to remove redundant candidate boxes. However, traditional methods rely heavily on manual design and feature selection, exhibit low efficiency and poor robustness, and are not capable of handling real-time autonomous driving. Deep learning architectures have emerged as two-stage and one-stage methods. Two-stage methods are based on convolutional neural networks for classification, of which Fast R-CNN is representative, with a high detection accuracy; however, it is still unable to eliminate the NMS process and cannot realize end-to-end detection (Ren et al., 2015). One-stage methods are based on convolutional neural networks for regression and perform better in terms of inference speed. Redmon et al. (2016) first proposed YOLO, and this series of algorithms (Ge et al., 2021; Li et al., 2023; Yung et al., 2022) occupies a dominant position among one-stage algorithms, with a wide range of industrial applications.

Transformer-based object detection methods have demonstrated significant application potential in autonomous driving technology. As the demands for adaptability to complex scenarios, real-time performance, and multimodal data processing in autonomous driving continue to increase, transformers, with their exceptional global modeling capabilities and end-to-end optimization framework, have become a key driving force in advancing perception technology for autonomous driving. In autonomous driving scenarios, challenges such as occlusion, lighting variations, and complex backgrounds are common. The global self-attention mechanism of transformers can accurately capture the global contextual information of the input data, thereby effectively separating objects from the background.

The architecture of the transformer, which is extensively used in natural language processing (NLP), has recently attracted interest in the field of computer vision (Vaswani et al., 2017). Carion et al. (2020) introduced the detection transformer (DETR), which reframes object detection as an ensemble prediction task, eliminating the need for NMS operations. This approach enables end-to-end object detection with enhanced global modeling capabilities, outperforming Fast R-CNN. In autonomous driving, objects are often occluded by buildings or other objects, DETR can infer the targets in occluded areas using global contextual information. Subsequently, Zhu et al. (2020) proposed Deformable DETR, which incorporates a deformable attention module to focus attention selectively on specific sampling points within the feature map, which reduces the computational overhead and accelerates training. Wang et al. (2022) presented the anchor DETR, integrating an anchor point mechanism into query vectors to address the issue of poor interpretability. DINO-DETR leverages a comparative training denoising method and hybrid query selection strategy for anchor point initialization (Zhang et al., 2022). Zong et al. (2023) introduced the H-DETR algorithm, which employs a hybrid matching approach during the Hungarian matching phase and incorporates one-to-many matching branches, offering a novel avenue for enhancement. BEVFormer (Li et al., 2024) achieves a more precise bird's-eye-view (BEV) environment modeling in complex scenarios, providing robust support for path planning and decision-making in autonomous driving systems. Mushtaq et al. (2024) proposed PLC-Fusion, which leverages transformer architectures to extract features from both images and point clouds, significantly enhancing the accuracy and efficiency of multimodal object detection.

Despite the diverse improvement perspectives provided by previous studies, in the field of autonomous driving, DETR-like detectors still suffer from the following problems:

The limited capability to detect objects across different scales, as well as lack of precision in determining the exact positions of objects, results in suboptimal detection accuracy in autonomous driving situations.

The performance of the model is hindered by the attention mechanism layer within the encoder, particularly when dealing with higher-resolution images. This results in a considerable increase in computational cost and memory complexity, which, in turn, affects both model accuracy and inference speed.

During the training phase, the hyperparameters, including the weight of each loss function, are manually set. However, this manual approach incurs a high cost for tuning the parameters and overlooks the dynamic balancing issue between the loss functions. Consequently, the model experiences slow convergence and lacks convergence accuracy.

Therefore, we propose an improved autonomous driving object detection method based on DETR, which contains three improvements:

• We propose a multi-scale feature and location information extraction method. A network that incorporates multi-scale residual partition units with a coordinate attention module was designed to improve the multi-scale detection and position sensing capabilities.

• We designed a transformer encoder based on an efficient attention mechanism. A grouped attention mechanism layer is deployed in the encoder, which computes the attention region in parallel in the horizontal and vertical groups, fully learns the image features from different directions, and maintains a balance between local and global information by controlling the range of attention computation. This effectively reduces the computational overhead and improves average precision (AP) and inference speed.

• We propose a novel dynamic hyperparameter tuning method based on Pareto efficiency, which involves automatically updating the weights of various loss functions during the training process, ensuring continuous coordination of their training states. The aim of this approach was to accelerate the convergence process and improve the accuracy of the final convergence of the detector.

### 1.1 Feature extraction in object detection

The feature extraction network utilizes multi-layered techniques to capture semantic information from images, focusing on two main approaches: convolutional neural network-based feature extraction network and transformer-based feature extraction network. Obtaining diverse object characteristics, particularly multi-scale features, not only speed up the convergence of the model but also greatly enhances the detection capability. ViT (Dosovitskiy et al., 2020), as a pioneering work to implement the transformer architecture in computer vision, has a larger receptive field and modeling capability than convolutional neural networks. When ViT is used as a feature extractor, it imposes a significant burden on model training. If multi-scale feature extraction is performed in such a case, this obviously expands on such drawbacks. DN-Deformable-DETR employs the Swin Transformer (Li et al., 2022), which has less computational overhead but still fails to capture multi-scale features at the corresponding stages.

In contrast, convolutional neural networks progressively deepen the extraction of features through convolution operators. This inherent property of extracting multi-scale features is advantageous for solving multi-scale feature problems. VGGNet uses stacked convolutional layers to solve multi-scale problems (Simonyan and Zisserman, 2014); however, the number of model parameters is large and inefficient. Lin et al. (2017) successfully deployed an FPN structure for object detection tasks; however, this approach seriously affects the inference speed and is even more inapplicable to the higher computational complexity of DETR-like models. DINO-DETR (Zhang et al., 2022) deploys convolution in the encoding and decoding phases; however, this increases the design difficulty of the model and significantly increases the amount of computation, which is not conducive to the development of a lightweight model. Recently, more efficient multi-scale feature extraction networks have emerged (Huang et al., 2017; Yu et al., 2018; Gao et al., 2019; Hou et al., 2021). Based on the characteristics of the object detection model, applying these networks in the backbone is a practical choice. In addition, current methods lack the ability to sense target location information (Hou et al., 2021), which is critical for improving the precision of object detection tasks. Building on previous research, our proposed network architecture focuses on extracting multi-scale features and precise location information to improve accuracy.

### 1.2 Transformer encoder

The transformer encoder is an essential part of the detector, and experiments have demonstrated that the encoder contributes approximately 11% to the AP but accounts for approximately 85% of the model's computational effort (Lin et al., 2022). The attention layer is the core of the encoder. It is more difficult for inefficient encoders to cope with autonomous driving object detection scenarios, which directly affects the inference speed.

DETR was the first to use the ViT module as an encoder, incorporating the transformer into the object detection framework by employing a multi-head attention (MSA) mechanism in the attention layer. MSA is a form of self-attention mechanism that converts a feature vector into a sequence, enabling the model to detect relationships between various components of the entire input through the representation of all possible interactions between elements within a sequence. In DETR, the global attention mechanism is computationally intensive, which leads to difficulties in model training. The Swin Transformer employs the idea of local attention, which limits attention computation to a fixed window and reduces the computational overhead (Liu Z. et al., 2021). On this basis, Shuffle Transformer further enhances the information exchange between windows over long distances by spatial shuffling (Huang et al., 2021), and CS Transformer employs “cross-shaped window attention” to improve computational efficiency (Dong et al., 2022). MobileVit employs a hybrid architecture that combines VIT and CNN for initial deployment on mobile devices (Mehta and Rastegari, 2021). ElasticViT first trains a high-quality VIT super-network and subsequently determines the best sub-network to be deployed to further reduce latency (Tang et al., 2023). In summary, this study focused on designing an efficient attention layer for encoders.

### 1.3 Optimization of model training parameters

During the training phase of an end-to-end network, multiple loss functions that handle both regression and classification tasks are commonly employed. However, it is often overlooked that these loss functions can interact with one another, significantly affecting model performance based on their relative weightings. Kendall et al. (2018) introduced a method of uncertainty to weigh losses by employing a Bayesian framework that emphasizes prediction uncertainty to automatically set weights for these loss functions. Mahapatra and Rajan (2020) enhanced the gradient-based multi-objective optimization algorithm by considering the loss function as multiple targets, assigning upper bounds to them, and successfully applying this optimization across different deep learning tasks. Lin et al. (2019) developed an algorithmic framework to ensure Pareto efficiency, thereby guaranteeing compliance with the Pareto condition and successful application of the optimization algorithm. Liu X. et al. (2021) introduced a novel gradient optimization algorithm using Stein variational gradient descent (SVGD) to analyze the Pareto frontier, which effectively solves high-dimensional problems and yields more uniformly distributed and diversified solutions on the Pareto front, thus optimizing the model. Lin et al. (2021) also proposed a random weighting approach, which includes both random loss and random gradient weighting and demonstrated improved generalization in experimental settings. These contributions present optimization strategies for model parameters from two major perspectives: the dynamic weighting of loss functions and gradients. However, these approaches are broad, and there has been limited research specifically targeting object detection models. Building on these advancements, this study focused on an algorithm designed to adjust the weights of the loss functions.

## 2 Improved object detection method for autonomous driving based on DETR

**Figure 1:**
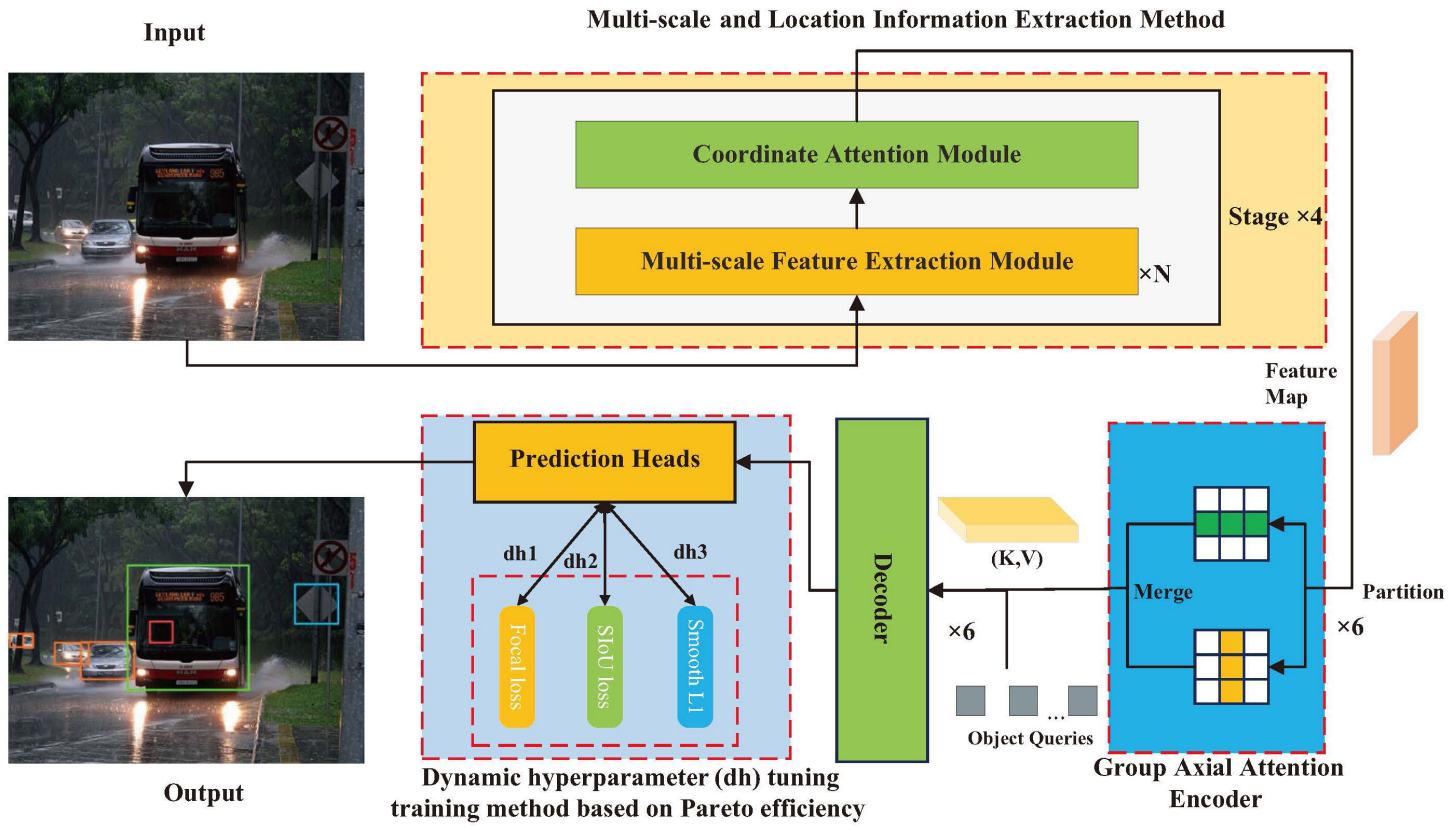
Overview the proposed method.

The method is composed of three parts, with the framework illustrated in Figure 1, including the multi-scale feature and location information extraction method, Transformer encoder based on the group axial attention mechanism, and dynamic hyperparameter tuning training method based on Pareto efficiency. To perform object detection for autonomous vehicle driving, the backbone network captures features from the image intended for detection and subsequently passes the feature map to the encoder. Following the encoding process of the feature map, vectors K and V are generated by the encoder and fed into the decoder in conjunction with the query vector Q. Finally, following the dynamic hyperparameter tuning training method based on Pareto efficiency, the prediction head captures the output of the decoder and ultimately derives the desired target information.

### 2.1 Multi-scale feature and location information extraction method

**Figure 2:**
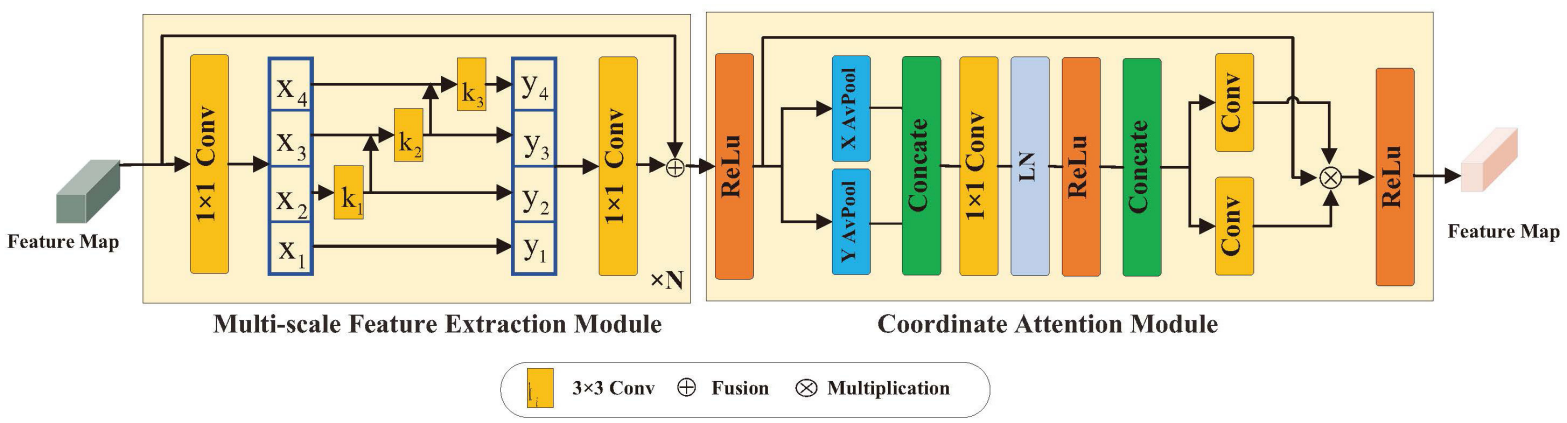
Multi-scale feature and location information extraction method.

Object detection is a crucial task that relies on both multi-scale features and precise location information of the target. Our research introduces a multi-scale feature and location information extraction method designed to acquire detailed features at multiple scales and enhance the target location information by improving the backbone network structure, as illustrated in Figure 2. The initial step involves passing the image input through a detection network consisting of four stages. Each stage comprises two types of modules: one for extracting multi-scale features and one for coordinating attention. At the beginning of each stage, the feature map is input into the multi-scale feature extraction module. This process integrates various residual units into the convolutional structure to extract features of different scales from the image. Subsequently, the feature map proceeds to the coordinate attention module, which captures the positional details from the extracted multi-scale features, further enhancing the detection capability.

#### 2.1.1 Multi-scale feature extraction module

The input feature map *X* ∈ *R*^*H*×*W*×*C*^ is partitioned into *n* groups after 1 × 1 convolution, and each group of data is represented by *X*_*i*_, where *i* ∈ {1, 2, ...*n*}, and the number of channels shrinks *n* minus a multiple into the residual unit branch, as shown in Figure 2. In each branch, except for *X*_1_, each group of data undergoes a 3 × 3 convolution operation, denoted as *K*_*i*_, and the output is denoted as *Y*_*i*_. *Y*_*i*_ is derived from the *i*-th group of data and the output *Y*_*i*−1_ of group *i* − 1 after. *Y*_*i*_ is defined as follows:


(1) $$\begin{array}{l} Y_{i}=\{\begin{array}{ll} X_{i} & i=1;\\ K_{i}\left(X_{i}\right) & i=2;\\ K_{i}\left(X_{i}+Y_{i-1}\right) & 2<i\leq \text{n}. \end{array} \end{array}$$

where the previously processed features *Y*_*i*−1_ are included in the convolution operation *Y*_*i*−1_ of the *i*-th group when *i* > 2. Segmentation is processed in a multi-scale manner, and each time the segmented feature *X*_*i*_ undergoes a 1 × 1 convolution, it expands the receptive field of *X*_*i*_. The outputs of all the different scales *Y*_*i*_, which are spliced in the channel dimension, are subjected to a 1 × 1 convolution operation to obtain a fused image, *Z*, which facilitates the extraction of the previous procedure, as shown in Equation 2:


(2) $$\begin{array}{l} Z=Conv\left(Concat\left(Y_{1},...,Y_{n}\right)\right) \end{array}$$

where *Y*_1_ is directly output without convolution, which serves to reuse the features. The methods described above enable the extraction of characteristics at a more precise level of granularity, featuring varied receptive fields and multiple scales. The above process achieves the progressive fusion of multi-scale features, where each set of convolutions relies on the output of the previous set. This step-by-step stacking mechanism captures features at different scales. The outputs of the *s* groups are aggregated through concatenation, forming a more powerful representation. Mathematically, this feature extraction process is similar to recursive convolution, ensuring that features are adequately expressed across different scales.

#### 2.1.2 Coordinate attention module

The module for coordinates exploits the location information in feature maps across channels, which not only aids in model recognition and localization of specific areas but also improves the detection of distant relationships in visual tasks. Figure 2 illustrates the use of an attention module that employs a network with branches to compute attention weights. These weights are subsequently multiplied with the initial feature map to generate the ultimate output. To process feature map *X* ∈ *R*^*H*×*W*×*C*^, adaptive mean pooling is conducted in the height and width directions. Combining features across spatial dimensions, these two operations produce a collection of direction-sensitive feature maps with dimensions *C* × *H* × 1 and *C* × 1 × *W*, respectively. Through these transformations, remote relationships are captured along one spatial dimension while maintaining precise position information along the other, as demonstrated in Equations 3, 4:


(3) $$\begin{array}{l} z_{c}^{h}\left(h\right)=\frac{1}{W}\underset{0\leq i\leq W}{\sum }X_{c}\left(h,i\right) \end{array}$$

(4) $$\begin{array}{l} z_{c}^{w}\left(w\right)=\frac{1}{H}\underset{0\leq i\leq H}{\sum }X_{c}\left(j,w\right) \end{array}$$

where $z_{c}^{h}\left(h\right)$ and $z_{c}^{w}\left(w\right)$ denote the outputs of the *C*-th channel for height *h* and width *w*, respectively. After the above operations, the position information is encoded, and the feature map is generated. First, the outputs of Equations 3, 4 are spliced and fed into a 1 × 1 convolution *F*_1_, as shown in Equation 5:


(5) $$\begin{array}{l} f=\delta \left(F_{1}\left[Z^{h},Z^{w}\right]\right) \end{array}$$

where [, ] denotes splicing by the spatial dimension, δ is the non-linear activation function, *f* ∈ *R*^*C*/*r*×(*H*+*W*)^ denote the feature maps generated in the horizontal and vertical directions, *f*^*h*^ ∈ *R*^*C*/*r*×*H*^ and *r* denotes the reduction multiplier. Subsequently, *f* ∈ *R*^*C*/*r*×(*H*+*W*)^ is divided into *f*^*h*^ ∈ *R*^*C*/*r*×*H*^ and *f*^*w*^ ∈ *R*^*C*/*r*×*W*^ by spatial dimension, and *f*^*h*^ and *f*^*w*^ are adjusted into tensors with the same number of channels using two 1 × 1 convolutions, as shown in Equations 6, 7:


(6) $$\begin{array}{l} g^{h}=\sigma \left(F_{h}\left(f^{h}\right)\right) \end{array}$$

(7) $$\begin{array}{l} g^{w}=\sigma \left(F_{w}\left(f^{w}\right)\right) \end{array}$$

where σ is the sigmoid function, *g*^*h*^ and *g*^*w*^ are the attention weights, and the output coordinate attention feature map is given by


(8) $$\begin{array}{l} Y_{c}\left(i,j\right)=X_{c}\left(i,j\right)\times g^{h}\left(i\right)\times g^{w}\left(j\right) \end{array}$$

### 2.2 Transformer encoder based on group axial attention mechanism

Although global attention mechanisms are effective in modeling long-range dependencies, they can result in high computational costs in downstream tasks, such as object detection, particularly with high-resolution images. Some approaches restrict attention to a window, which can hinder the information exchange between windows and limit the receptive field. Therefore, we introduce a transformer encoder that utilizes a group axial attention layer. Unlike in traditional transformer encoders, the input features in the group axial attention layer are divided into horizontal and vertical groups based on their dimensions. Subsequently, self-attention is calculated separately within each group before merging and mapping to the output. This approach allows for the comprehensive learning of image features from various orientations while maintaining a balance between local and global information by controlling the attention range. As a result, it effectively reduces computational costs and enhances inference speed and accuracy.

#### 2.2.1 Structure of the encoder

**Figure 3:**
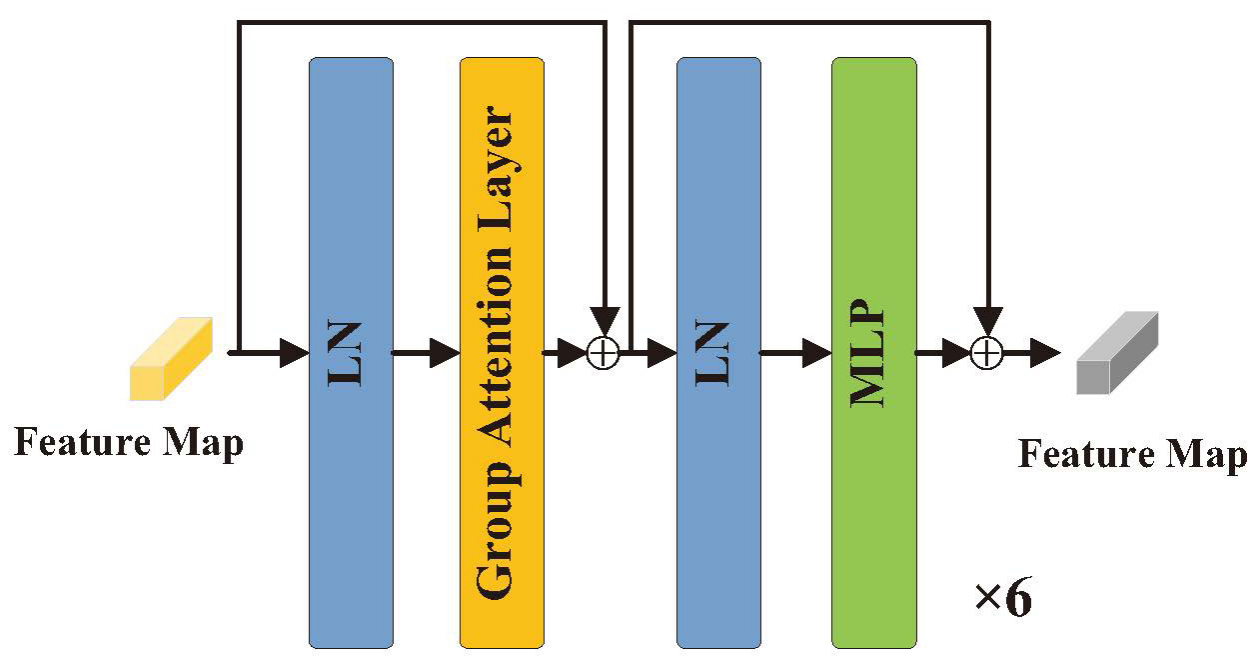
Structure of encoder.

As illustrated in Figure 3, the encoder consists of six identical layers for the input *T* ∈ *R*^*H*×*W*×*C*^, which is transformed into a matrix *T* ∈ *R*^*N*×*C*^. Before entering the encoder, the structure is represented by


(9) $$\begin{array}{l} \{\begin{array}{l} Z_{L}^{\prime }=T_{L-1}+GL\left(T_{L-1}\right)\\ Z_{L}=LN\left(Z_{L}^{\prime }\right)+FFN\left(Z_{L}^{\prime }\right),L=1,2,\ldots ,6\\ Y=Z_{6} \end{array} \end{array}$$

where *LN*(), *FFN*(), and *GL*() denote layer normalization, feed-forward neural network, and group axial attention layer, respectively *T*_*L*−1_ denotes the output of the layer *L* − 1 encoder; $Z_{L}^{\prime }$ and *Z*_*L*_ denote the results from the layer *L* encoding process; and *Y* ∈ *R*^*N*×*C*^ denotes the output of the layer 6 encoder, i.e., the final encoding result for the feature map *T* ∈ *R*^*H*×*W*×*C*^.

#### 2.2.2 Group axial attention layer

**Figure 4:**
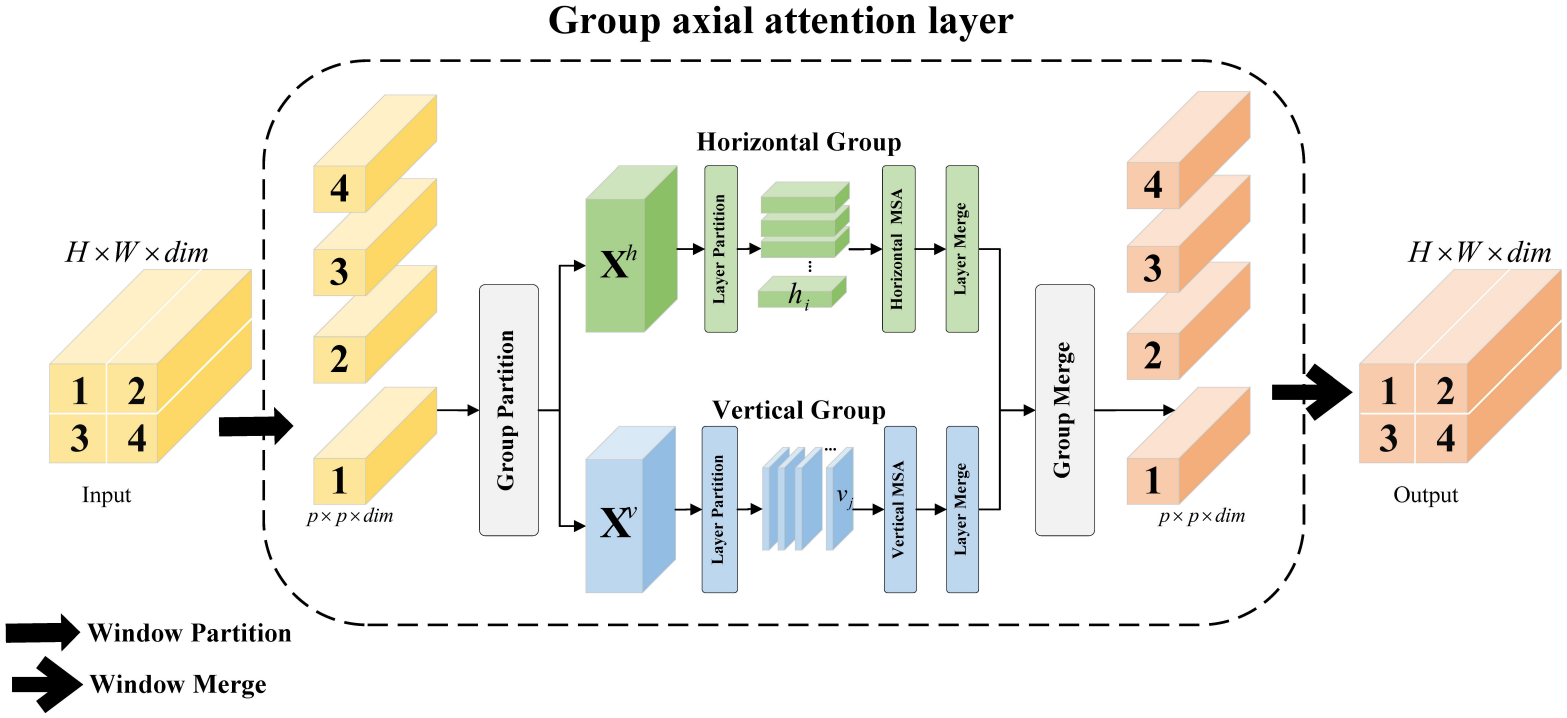
Illustration of GA and WGA.

As shown in Figure 4, for the input feature vector *X* ∈ *R*^*H*×*W*×*C*^, *H* and *W* denote the height and width, respectively. *Liner*1 maps the channel dimension *C* to *dim*, yielding *X* ∈ *R*^*H*×*W*×dim^, which serves as the input to group axial attention layer. The hyperparameter *K* is set to the number of heads, and *C* is divided into two parts, *X*^*h*^ ∈ *R*^*H*×*W*×dim/2^ and *X*^*v*^ ∈ *R*^*H*×*W*×dim/2^, according to the *dim*, representing the horizontal and vertical groups, respectively. In the horizontal group, the length of the horizontal layer *h*_*i*_ is set to *s*, and *h*_*i*_ is $\frac{W}{s}$. Similarly, the number of vertical layers *j* is $\frac{H}{s}$. In each horizontal layer *h*_*i*_, $h_{i}\in R^{s\times W\times \dim/2}$ is serialized as a vector $\eta_{i}\in R^{sW\times \dim/2}$, and *K*/2 heads are set for the computation of the multi-head attention. The vertical group divides the vertical layer *v*_*j*_ by *s* and performs the same operation. The horizontal and vertical groups are spliced, and *dim* is mapped back to *C* by the *Liner*2. The group axial attention computation process is shown in Equation 10:


(10) $$\begin{array}{l} \{\begin{array}{c} X=Liner1\left(X\right)\\ X^{h},X^{v}=Spilt\left(X\right)\\ \left[h_{1},h_{2},...,h_{i}\right]=Spilt\left(X^{h}\right),\left[v_{1},v_{2},...,v_{j}\right]=Spilt\left(X^{v}\right)\\ H-Attention=Concat\left[MSA\left(h_{1}\right)MSA\left(h_{2}\right),...,MSA\left(h_{i}\right)\right]\\ V-Attention=Concat\left[MSA\left(v_{1}\right)MSA\left(v_{2}\right)\text{,}..\text{,}MSA\left(v_{j}\right)\right]\\ G\;-Attention\;=\;Concat\left[V-Attention,\;H-Attention\right]\\ \text{X}=\;Liner2\left(\;G-Attention\right) \end{array} \end{array}$$

The group axial attention layer combines *H* − *Attention* and *V* − *Attention* in the channel dimension to create group axial attention. This layer maintains the feature map dimensions while evenly distributing the heads into different attention groups. This distribution helps capture various features and patterns in image sequences, ultimately improving the model's expressive and generalization capabilities. In the horizontal and vertical groups, the range of attention can be changed by adjusting *s*. Experiments have shown that the best accuracy is achieved when *s* = [1, 1, 2, 2, 6, 6].

#### 2.2.3 Analysis and variants

Our approach differs from traditional attention mechanisms in that it captures extensive features and manages the attention scope by separating horizontal and vertical layers. This method effectively reduces the computational complexity. When computing the group axial attention, the computational overhead of operations such as division is negligible, whereas the computation of the two branches is parallel. As shown in Figure 4, in each branch, features are learned from the subspace divided between multi-layer blocks, and mappings are added to the output to further enhance the encoder.

This section further explores the relationship between the attention mechanism and computational complexity. Assuming that the feature map size to be processed is *H* × *W* × *C*, the computational complexity is given by Equation 11 when using the multi-head attention mechanism (MSA). The attention range is set to s. When group axial attention (GA), the computational complexity is given by Equation 12. On this basis, Window G-Attention (WGA) is designed, and only the operations of splitting and merging windows are added, as shown in Figure 4, where the size of the window is set as *p* × *p*, and calculation of the group axial attention is performed in a fixed window, as shown in Equation 13:


(11) $$\begin{array}{l} \Omega \left(MSA\right)\;=HWC*\left(4C+2HW\right) \end{array}$$

(12) $$\begin{array}{l} \Omega \left(GA\right)\;=HWC*\left(4C+sH+sW\right) \end{array}$$

(13) $$\begin{array}{l} \Omega \left(WGA\right)\;=HWC*\left(4C+2sp\right) \end{array}$$

In practice, the computational volume increases sharply when *H* and *W* increase. *s* is usually much smaller than *H* and *W*, which requires less attention computation. In the calculation of WGA, *H* = *W* is adjusted, and the operation of dividing the window is performed first. The window size is *p* × *p*, which further reduces the amount of computation and facilitates faster inference. The value of *s* should be appropriately adjusted as the encoders continue to stack. To reduce the limitation of window size on WGA, the value of *p* should not be too small. The core principle of this approach is to divide the input feature map into multiple windows and compute local self-attention separately within the horizontal and vertical directions. The results are then concatenated and fused through linear projection to extract both local and global features. This significantly reduces computational complexity, making it efficient and suitable for high-resolution images.

### 2.3 Dynamic hyperparameter tuning training method based on Pareto efficiency

**Figure 5:**
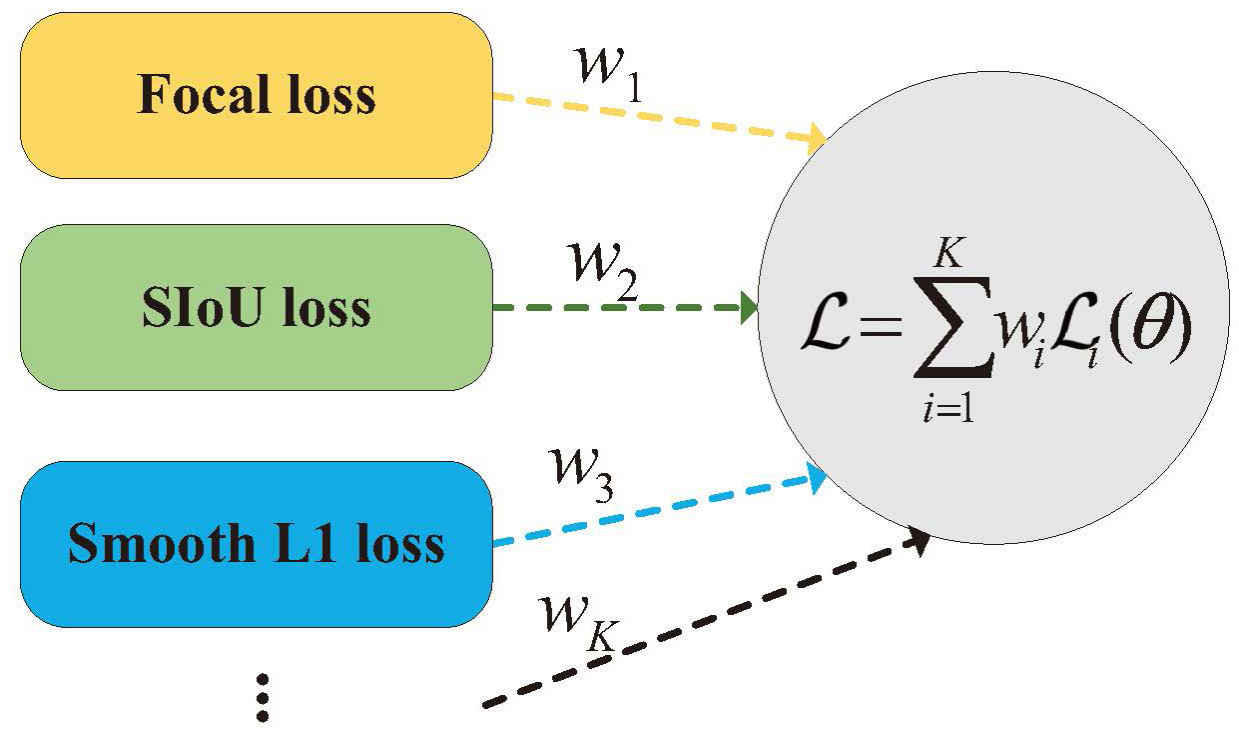
Illustration of functions and algorithm.

In the model training stage, different classes of loss functions are typically utilized, including intersection over union (IoU), classification, and localization losses, as illustrated in Figure 5. This study employed the SIoU loss function to enhance the regression efficiency by guiding the direction of the prediction box toward the ground truth box (Rezatofighi et al., 2019). For classification loss, the focal loss was employed, while Smooth-L1 loss was used for localization loss. The weights of the loss functions are essential for balancing the various components to improve the training outcomes, particularly in object detection tasks. Traditionally, manual methods are used to set fixed hyperparameters as weights for weight assignment. Nevertheless, this strategy might disregard the possibility that the complexity of training different loss functions could vary over the course of training, potentially restricting the learning capacity. To address this issue, a dynamic hyperparameter tuning training method based on Pareto efficiency is introduced in this section. This method treats loss functions as multiple interacting objectives and continuously optimizes them by dynamically weighting and coordinating these objectives, thereby accelerating the convergence process and enhancing accuracy at convergence.

#### 2.3.1 Introduction to loss functions

##### 2.3.1.1 SIoU loss

###### 2.3.1.1.1 Angle cost

**Figure 6:**
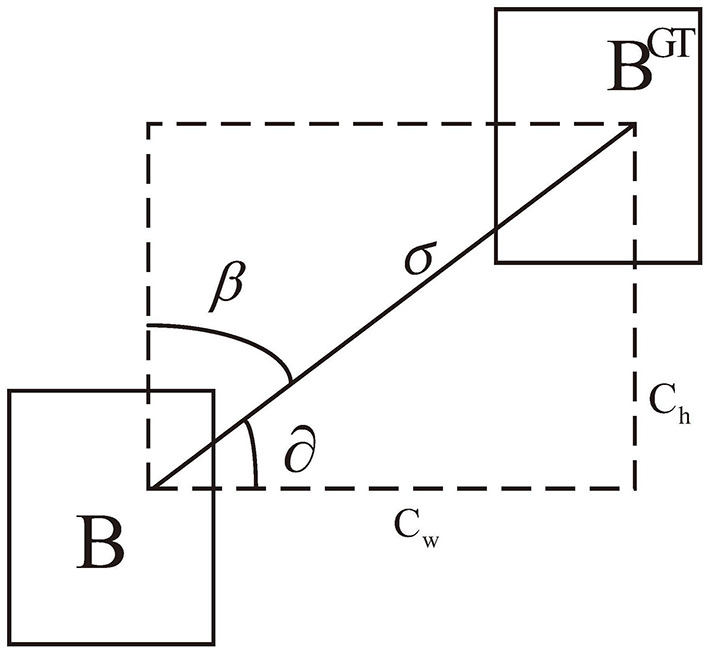
Illustration of angle cost.

Angle loss plays a crucial role in expediting the convergence process before the prediction box and ground truth box are matched. This is achieved by initially regulating the angle factor to align them on the same horizontal or vertical line. As shown in Figure 6, the loss function minimizes ∂ when $\partial <\frac{\pi }{4}$. Otherwise, it minimizes $\beta =\frac{\pi }{2}-\partial$. The angular loss is defined as


(14) $$\begin{array}{l} \Lambda =1-2*\sin^{2}\left(\arcsin\left(x\right)-\frac{\pi }{4}\right) \end{array}$$

where $x=\frac{c_{h}}{\sigma }=\sin\left(\partial \right)$, $\sigma =\sqrt{{\left(b_{c_{x}}^{gt}-b_{c_{x}}\right)}^{2}+{\left(b_{c_{y}}^{gt}-b_{c_{y}}\right)}^{2}}$, and $c_{h}=max\left(b_{c_{y}}^{gt}-b_{c_{y}}\right)-min\left(b_{c_{y}}^{gt}-b_{c_{y}}\right)$.

###### 2.3.1.1.2 Distance cost

**Figure 7:**
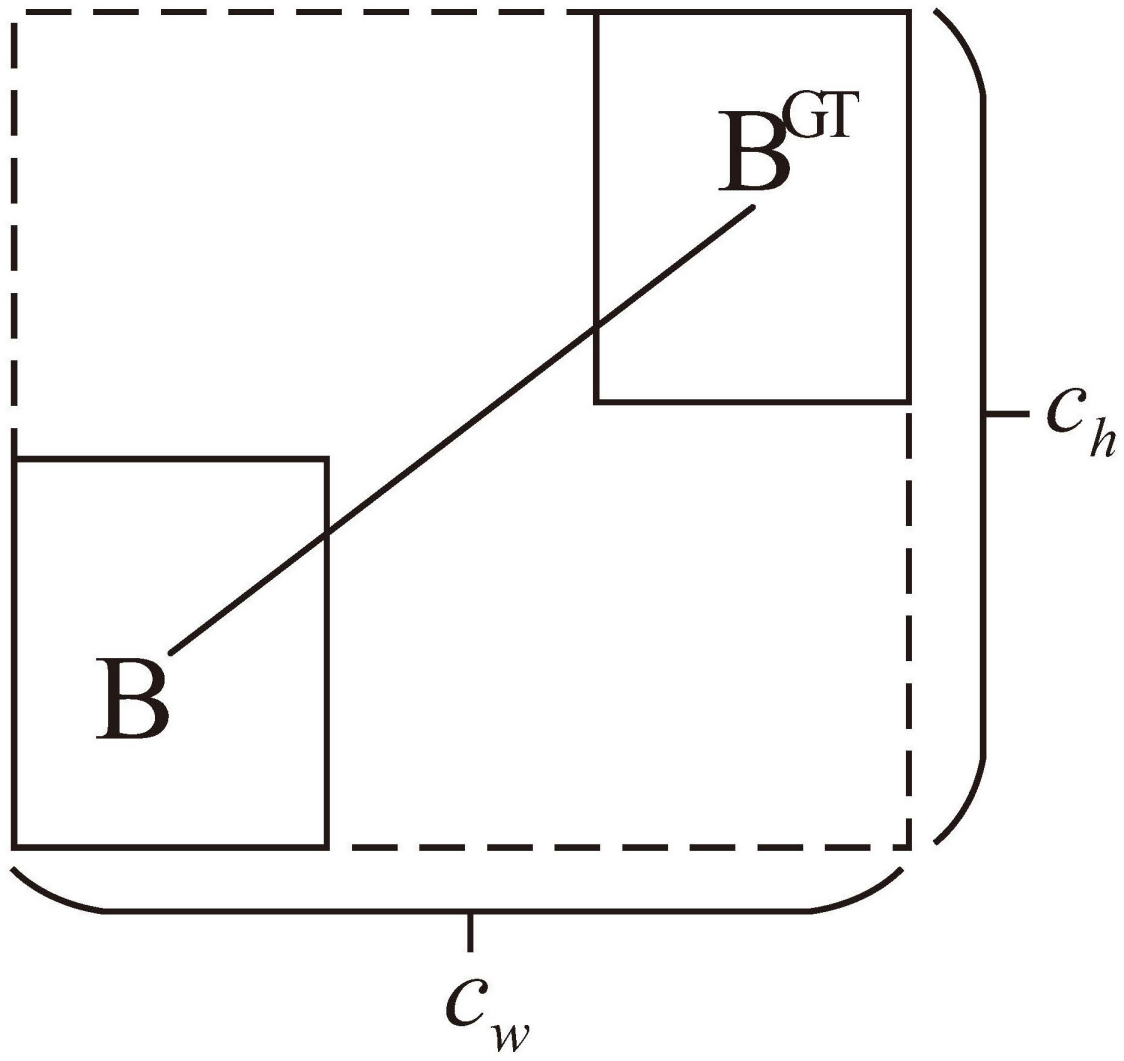
Illustration of distance cost.

As demonstrated in Figure 7, when the prediction box and ground truth box are aligned either horizontally or vertically but remain significantly apart, it is crucial to impose a constraint on their separation distance. Building on the angle cost, the distance cost is defined by Equations 15, 16:


(15) $$\begin{array}{l} \Delta =\underset{t=x,y}{\sum }\left(1-e^{-\gamma \rho_{t}}\right) \end{array}$$

(16) $$\begin{array}{l} \rho_{x}={\left(\frac{b_{c_{x}}^{gt}-b_{c_{x}}}{c_{w}}\right)}^{2},\rho_{y}={\left(\frac{b_{c_{y}}^{gt}-b_{c_{y}}}{c_{h}}\right)}^{2},\gamma =2-\Lambda \end{array}$$

where the width and height of the outer rectangle are represented by *c*_*w*_ and *c*_*h*_ for the prediction box and ground truth box, respectively. γ is utilized to regulate the impact of the angular loss on the distance cost.

###### 2.3.1.1.3 Shape cost

Shape loss describes the similarity between the shapes of the prediction box and ground truth box, as defined in Equations 17, 18:


(17) $$\begin{array}{l} \Omega =\underset{t=w,h}{\sum }{\left(1-e^{-\omega_{t}}\right)}^{\theta } \end{array}$$

(18) $$\begin{array}{l} \omega_{w}=\frac{\left|w-w^{gt}\right|}{\max\left(w,w^{gt}\right)},\omega_{h}=\frac{\left|h-h^{gt}\right|}{\max\left(h,h^{gt}\right)} \end{array}$$

where (*w, h*) and (*w*^*gt*^, *h*^*gt*^) denote the width and height of the ground truth box and prediction box, respectively. The value of θ indicates the degree of shape control, where a smaller θ indicates a higher degree of control; typically, θ ∈ [2, 6].

###### 2.3.1.1.4 IoU cost

The IoU quantifies the extent of overlap between the predicted bounding box and actual ground truth box. IoU loss is defined as follows:


(19) $$\begin{array}{l} \text{IoU}=\frac{\left|B\;\cap \;B^{GT}\right|}{\left|B\;\cup \;B^{GT}\right|} \end{array}$$

where *B*∩*B*^*GT*^ denotes the overlapping area of the prediction box and ground truth box, and *B*∪*B*^*GT*^ denotes the concurrent area of the prediction box and ground truth box. SIoU loss is defined by Equation 20:


(20) $$\begin{array}{l} L_{\text{siou}}=1-IoU+\frac{\Delta +\Omega }{2} \end{array}$$

##### 2.3.1.2 Focal loss

Focal loss aims to enhance the focus on challenging samples by reducing the weight of easy-to-classify samples and amplifying the weight of difficult-to-classify samples, as defined in Equations 21, 22:


(21) $$\begin{array}{l} p_{t}\{\begin{array}{c} p,y=1\\ 1-p,others \end{array}\; \end{array}$$

(22) $$\begin{array}{l} Focalloss\left(p_{t}\right)=-\alpha_{t}{\left(1-p_{t}\right)}^{\gamma }\log\left(p_{t}\right)\; \end{array}$$

where *p* represents the probability of the model output, *y* denotes the true label, and γ is the weight factor.

##### 2.3.1.3 Smooth-L1 loss

The Smooth-L1 loss function combines the advantages of the L1 and L2 losses, with a smooth and robust training process, defined as


(23) $$\begin{array}{l} L_{\text{Smooth}-L1}\left(b_{i}^{gt},{\hat{b}}_{i}\right)=\{\begin{array}{c} \frac{1}{2}\sum_{i=0}^{N}{\left(b_{i}^{gt}-{\hat{b}}_{i}\right)}^{2},\text{if}|b_{i}^{gt}-{\hat{b}}_{i}|<1\\ \sum_{i=0}^{N}|b_{i}^{gt}-{\hat{b}}_{i}|-0.5,\text{other} \end{array} \end{array}$$

where $b_{i}^{gt}$ denotes the position coordinates of the *i*-th ground truth box, and ${\hat{b}}_{i}$ denotes the position coordinates of the *i*-th prediction box.

##### 2.3.1.4 Overall loss function

In summary, the function is defined as


(24) $$\begin{array}{l} L=w_{1}L_{cls}+w_{2}L_{\text{siou}}+w_{3}L_{Smooth-L1} \end{array}$$

where *L*_cls_ denotes the category focal loss, *L*_siou_ denotes SIoU loss, *L*_*Smooth*−*L*1_ denotes Smooth-L1 loss, and *w*_1_, *w*_2_, *w*_2_ and are the respective weight parameters.

#### 2.3.2 Algorithm of dynamic hyperparameter tuning training method based on Pareto efficiency

##### 2.3.2.1 Pareto efficiency and loss functions

Pareto efficiency is a concept in multi-objective optimization. To minimize a set of objective functions *f*_1_, ..., *f*_*K*_ in a given system, Pareto efficiency is a state in which it is impossible to improve one objective without hurting others.

Definition 1. To minimize objectives, denote the outcomes of two solutions by $s_{i}=\left[f_{1}^{i},...,f_{K}^{i}\right]$ and $s_{j}=\left[f_{1}^{j},...,f_{K}^{j}\right]$, where *s*_*i*_ dominates if and only if $f_{1}^{i}\leq f_{1}^{j},f_{2}^{i}\leq f_{2}^{j},...,f_{K}^{i}\leq f_{K}^{j}$.

Definition 2. A solution $s_{i}=\left[f_{1}^{i},...,f_{K}^{i}\right]$ is Pareto efficient if no other solution $s_{j}=\left[f_{1}^{j},...,f_{K}^{j}\right]$ dominates *s*_*i*_.

Our goal is to discover Pareto efficient solutions. It is important to recognize that these solutions are not unique, leading to the establishment of the Pareto frontier. In summary, the training of object detection loss functions can be viewed as an optimization task involving the minimization of multiple loss functions. As illustrated in Figure 5, a set of weights, denoted as *w*, is determined to enable Pareto efficiency to be achieved by the loss functions. By iteratively solving for these weights *w* during the training process, we can continually and effectively optimize the objectives.

##### 2.3.2.2 Conditions of the algorithm

It is assumed that there are *K* differentiable loss functions L_*i*_(θ), where θ denotes the model parameters in the object detection model *F*(θ), ∀*i* ∈ {1, ..., *K*}. The *K* loss functions correspond to the *K* objectives to be optimized, and multiple objectives are merged into a single one by setting a scalarization weight for the objectives, as shown in Equation 25:


(25) $$\begin{array}{l} L\left(\theta \right)=\overset{K}{\underset{i=1}{\sum }}\omega_{i}L_{i}\left(\theta \right) \end{array}$$

where $\overset{K}{\underset{i=1}{\sum }}\omega_{i}=1$, ω_*i*_ ≥ 0, ∀*i* ∈ {1, ..., *K*}. Boundary constraints of ω_*i*_ on *c*_*i*_ are set as ω_*i*_ ≥ *c*_*i*_, $\overset{K}{\underset{i=1}{\sum }}c_{i}\leq 1$,*c*_*i*_ ∈ [0, 1], ∀*i* ∈ {1, ..., *K*}.

To obtain Pareto efficient solutions, the aggregated objective loss function must be minimized, and the model parameters should satisfy the KKT (Chen, 2022) condition such that Equations 26, 27 are satisfied:


(26) $$\begin{array}{l} \overset{K}{\underset{i=1}{\sum }}\omega_{i}=1,\exists \omega_{i}\geq c_{i},i\in \left\{1,...,K\right\} \end{array}$$

(27) $$\begin{array}{l} \overset{K}{\underset{i=1}{\sum }}\omega_{i}\nabla_{\theta }L_{i}\left(\theta \right)=0 \end{array}$$

where ∇_θ_L_*i*_(θ) represents the gradient of L_*i*_. Considering the specific problem to be solved, we transform the KKT condition is as follows:


(28) $$\begin{array}{l} \min.\|\overset{K}{\underset{i=1}{\sum }}\omega_{i}\nabla_{\theta }L_{i}\left(\theta \right){\|}_{2}^{2}\text{s}.\text{t}.\overset{K}{\underset{i=1}{\sum }}\omega_{i}=1,\omega_{i}\geq c_{i},\forall i\in \left\{1,\ldots ,K\right\} \end{array}$$

A solution satisfying Equation 28 is a Pareto efficient solution. It has been demonstrated that these solutions result in gradient directions that minimize all loss functions (Sener and Koltun, 2018).

##### 2.3.2.3 Framework of the algorithm

The framework begins with a uniform scalarization weight and proceeds by alternately updating the weights and model parameters. An optimizer is then utilized to ensure that the model converges effectively. As shown in Table 1 and Algorithm 1, the key part of the algorithm is to solve for conditionally generated weights for Pareto efficiency.

**Table 1:** Notations and description.

**Notations**	**Description**
*F*(θ)	The object detection model
θ	The model parameters
L_*i*_(θ)	The loss function of for *i*-th objective
ω_*i*_	The weight of *i*-th objective for scalarization
*c*_*i*_	The boundary constraint for *i*-th objective
∇_θ_L_*i*_(θ)	The gradient of loss L_*i*_(θ)with respect of θ
*G*	The stacking matrix of ∇_θ_L_*i*_(θ)
*e*	The vector whose elements are all 1

**Algorithm 1:**
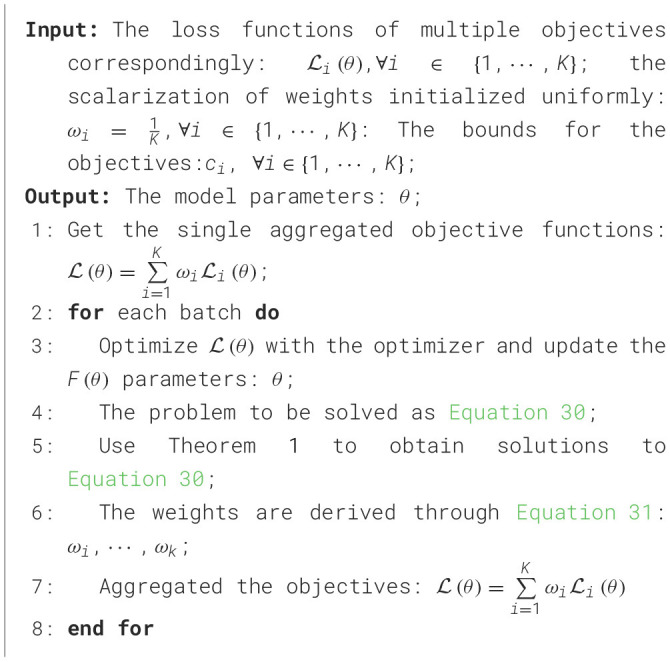
Dynamic hyperparameter tuning training method based on Pareto efficiency.

According to Equation 28, the problem is transformed into a quadratic programming algorithm by denoting ${\hat{\omega }}_{i}$ as ω_*i*_ − *c*_*i*_, and the Pareto efficiency condition becomes


(29) $$\begin{array}{l} \min.\|\overset{K}{\underset{i=1}{\sum }}\left({\hat{\omega }}_{i}+c_{i}\right)\nabla_{\theta }L_{i}\left(\theta \right){\|}_{2}^{2}\text{s}.\text{t}.\overset{K}{\underset{i=1}{\sum }}{\hat{\omega }}_{i}=1-\overset{K}{\underset{i=1}{\sum }}c_{i} \end{array}$$

The Pareto efficiency condition is equivalent to Equation 29. However, addressing the issue at hand is not straightforward, given its quadratic programming structure. Initially, we opt to ease these limitations by focusing solely on the equation restrictions. Subsequently, we implement a projection technique that produces effective outcomes from the viable set that encompassed all restrictions. When all other constraints are omitted except for the equational constraints, as shown in Equation 30, the solution is given by Theorem 1.


(30) $$\begin{array}{l} \min.\|\overset{K}{\underset{i=1}{\sum }}\left({\hat{\omega }}_{i}+c_{i}\right)\nabla_{\theta }L_{i}\left(\theta \right){\|}_{2}^{2}\text{s}.\text{t}.\overset{K}{\underset{i=1}{\sum }}{\hat{\omega }}_{i}=1-\overset{K}{\underset{i=1}{\sum }}c_{i} \end{array}$$

THEOREM 1. The solution to Equation 30 is ${\hat{\omega }}^{*}=\left({\left(M^{⊤}M\right)}^{-1}M\tilde{z}\right)\left[1:K\right]$ where *G* ∈ *R*^*K*×m^ is the stacking matrix of ∇_θ_L_*i*_(θ), *e* ∈ *R*^*K*^ is the vector whose elements are all 1, *C* ∈ *R*^*K*^ is the concatenated vector of *c*_*i*_, and $\tilde{Z}\in R^{K}$ is the concatenated vector of −*GG*^⊤^*c* and $1-\overset{K}{\underset{i=1}{\sum }}c_{i}$ and $M=\left(\begin{array}{cc} GG^{⊤} & e\\ e & 0 \end{array}\right)$.

During the solution process, the inverse operation of the matrix is negligible because the number of loss functions for object detection is small. However, the solution ${\hat{\omega }}^{*}$ of Equation 30 may be invalid because the non-negative constraints are ignored. Therefore, we obtain an effective solution using the projection method, as shown in Equation 31:


(31) $$\begin{array}{l} \min.\|\tilde{\omega }-{\hat{\omega }}^{*}{\|}_{2}^{2}\text{s}.\text{t}.\overset{K}{\underset{i=1}{\sum }}{\hat{\omega }}_{i}=1,{\tilde{\omega }}_{i}\geq 0,\forall i\in \left\{1,...,K\right\} \end{array}$$

Equation 31 represents a non-negative least squares problem, which can be easily solved using the active set method (Arnström and Axehill, 2021).

### 2.4 Summary

In the previous section, we presented three aspects of the improvement approach for unmanned object detection. First, we introduced an architecture to solve the insufficiency of the model for multi-scale object localization and detection. Subsequently, we developed a transformer encoder with group axial attention to reduce computation and enhance the inference speed. Finally, we presented a novel training technique that utilizes dynamic hyperparameter tuning inspired by the principle of Pareto efficiency. By dynamically adjusting the weights to align the training states of different loss functions, this approach effectively addresses issues related to manually assigning fixed weights. As a result, it enhances both the speed and accuracy of model convergence.

## 3 Experimental results and analysis

### 3.1 Setups

#### 3.1.1 Dataset

Dataset 1 was selected from the COCO 2017 (Lin et al., 2014) dataset with category objectives related to autonomous driving, consisting of 10 categories, 35,784 images for training, and 2431 images for validation. Dataset 2 combines the original categories from the PASCAL VOC 2012 (Everingham et al., 2010) dataset, which include Person, Car, Train, Motorcycle, Bicycle, and Other, with 11,540 images for training and 2913 images for validation. Dataset 3 is sourced from the KITTI professional autonomous driving dataset (Geiger et al., 2013), primarily including categories such as Car, Pedestrian, Cyclist, Van, Truck, and Tram, with 7,481 images for training and 7,518 images for validation.

#### 3.1.2 Evaluation metrics

The experimental evaluation metrics were AP, FPS, and GFLOPs. FPS and GFLOPs denote the inference speed and computation of the model, respectively. Specifically, AP_S_ and AP_M_ denote the AP for small- and medium-sized objects, respectively. AP is the area under the precision-recall (PR) curve, and precision (P) and recall (R) are calculated using Equations 32, 33:


(32) $$\begin{array}{l} P=\frac{TP}{TP+FP} \end{array}$$

(33) $$\begin{array}{l} R=\frac{TP}{TP+FN} \end{array}$$

where TP, FP, and FN denote the accurately recognized positive samples, erroneously recognized positive samples, and erroneously recognized negative samples, respectively. Mean average precision (mAP) can be computed by taking the average of the AP values across different categories, as illustrated in Equations 34, 35:


(34) $$\begin{array}{l} AP=\int_{0}^{1}P\left(R\right)dR\; \end{array}$$

(35) $$\begin{array}{l} mAP=\frac{1}{n}\overset{n}{\underset{i=1}{\sum }}AP_{i} \end{array}$$

In the context of object detection, the average precision (AP) is calculated across different Intersection over Union (IoU) thresholds ranging from 0.5 to 0.95 with increments of 0.05. When the IoU threshold is specifically set at 0.5, it is denoted as AP_50_. The mAP is then computed as the average of the AP values for each category in the dataset. Therefore, all individual AP values mentioned correspond to the overall mAP.

#### 3.1.3 Implementation details

The model was trained on an NVIDIA V100 GPU. In each stage the multi-scale feature and location information extraction method (MLEM), *N* was set to 3, 4, 6, and 3 correspondingly. The Adam optimizer was used for all experiments: initial_learning_ rate = 0.0005, weight_decay = 0.0001, and batchsize = 8.

### 3.2 Analysis of model parameters

#### 3.2.1 Parameter analysis of multi-scale feature extraction module

**Figure 8:**
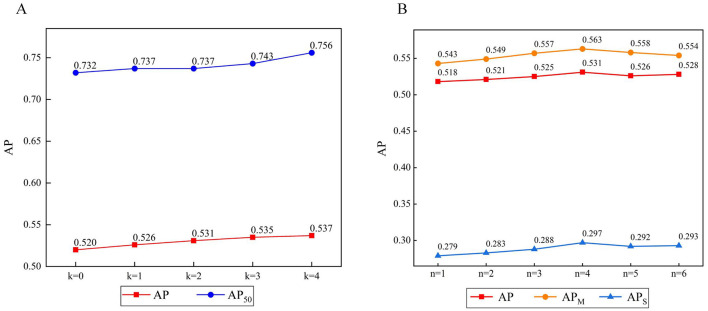
**(A)** The result of the number of CA modules. **(B)** The result of the number of scales.

To explore the effect of the scale *n* on this approach, *n* was set as a scale control parameter. Six experiments were designed with *n* = 1, 2, 3, 4, 5, 6. *n* = 1 represents a scale of one with no multi-scale fusion. Similarly, *n* = 2 represents a scale of 2, and the data are divided into two parts for multi-scale fusion. We adopted AP, AP_M_, and AP_s_ as indicators, as shown in Figure 8.

The experimental results show that the multi-scale fusion operation has a significant effect on AP_S_. An increase of *n* indicates that the number of different scales of the feature map fusion increases, and all show an upward trend. When *n* = 4, the multi-scale effect is obvious and optimal. When *n* = 5 or 6, the value of AP decreases slightly. Probably owing to limitations on the image size, the multi-scale feature extraction ability remains almost unchanged. However, an excessive number of branches can lead to a model degradation.

#### 3.2.2 Parameter analysis of coordinate attention module

To investigate the effects of the CA module on the experiments, *k* was set as the experimental parameter. Five experiments were designed with *k* = 0, 1, 2, 3, 4. There are four stages: *k* = 0 denotes no use of CA, *k* = 1 denotes that the module is deployed in stage 1, *k* = 2 denotes that the module is deployed in the first two stages, etc. We adopted AP as an indicator, as shown in Figure 8.

The experimental results show that the CA module effectively improves the AP compared with the case of *k* = 0. Building on the multi-scale feature maps obtained in the previous stages helps further improve the effectiveness of CA in later stages. When *k* = 1 or 2, shallow features, such as space and details, are retained, which helps improve the AP. When *k* = 4, the enhancement effect of AP is weakened, but AP reaches its peak.

#### 3.2.3 Parameter analysis of group axial attention layer

**Figure 9:**
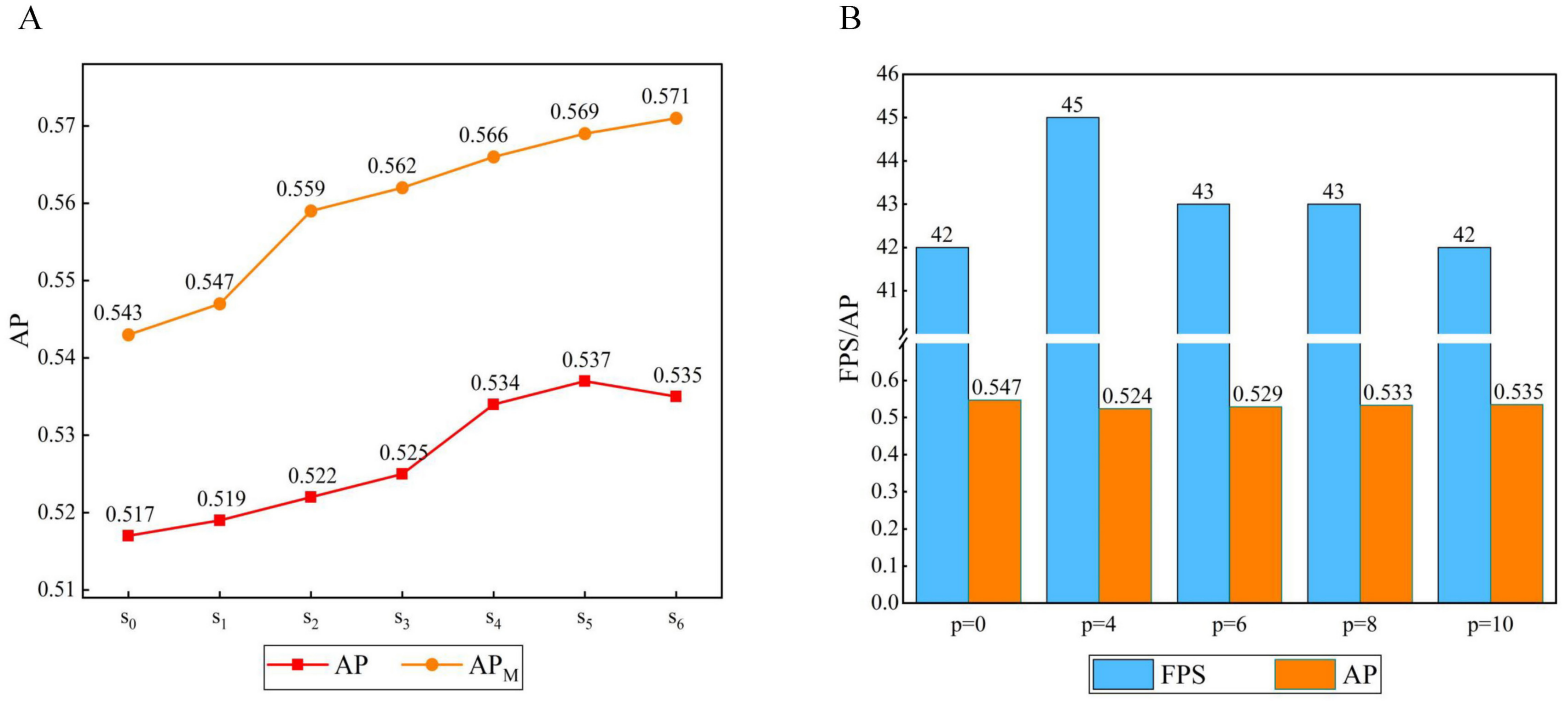
**(A)** Result of the range of group axial attention layer. **(B)** Result of the window size of window axial group attention.

To investigate the effect of the attention range *s* on the feature map in the group axial attention layer, we set the number of encoders *n* = 6, the attention layer in the encoder adopts a single attention range for each encoder to the feature map, *s*_*i*_ denotes the *i*-th range combination, *s*_0_ denotes the original method, *s*_1_ = [1, 1, 1, 1, 1, 1], *s*_2_ = [2, 2, 2, 2, 2, 2], *s*_3_ = [1, 1, 1, 2, 2, 2], *s*_4_ = [1, 1, 2, 2, 4, 4], *s*_5_ = [1, 1, 2, 2, 6, 6], *s*_6_ = [2, 2, 4, 4, 6, 6], and we performed seven experiments, as shown in Figure 9.

When *s*_0_ becomes *s*_1_, the AP gradually increases. When the attention range is *s*_2_ the accuracy is further improved, probably due to the expansion of the attention calculation range. As the encoders continue to stack, the image feature level continuously increases. However, *s*_1_ and *s*_2_ do not take this case into account. From *s*_3_ to *s*_6_, the attention range increases. In the early stages, the smaller attention range facilitates learning of the local details of the image. In the later stages, a larger range is more conducive to learning the global information of the image. Therefore, *s*_3_ from to *s*_5_, AP_M_ is significantly improved compared to the cases of *s*_1_ and *s*_2_, and the AP reaches its best for *s*_5_. Comparing *s*_6_ and *s*_1_, it can be observed that a larger attention range is more favorable for targets at the medium scale.

#### 3.2.4 Parameter analysis of window group axial attention

To explore the effect of window size on window group axial attention, we set the window size *p* as the experimental parameter, adjusted the size of the feature map to *W* = *H* in the encoder group axial attention layer, set the number of encoders to *n* = 6, and the attention range of the feature map in the group axial attention layer in each encoder was set to *s*_5_ = [1, 1, 2, 2, 6, 6], where *p* = 0 denotes no window partition, *p* = 1 denotes a partition size of 1 × 1, etc. Five experiments were conducted, as shown in Figure 9.

It follows that dividing the attention range within a fixed window can further reduce the computational complexity. When *p* = 4, the window size is smaller, the computation complexity is minimized, and the FPS increases; however, this will have a greater impact on dividing the attention region, which will lead to a lower AP. When p is larger, the impact on the operation of dividing the attention region is reduced, and although the individual window complexity increases, it leads to further increases in FPS and AP.

### 3.3 Ablations

Ablation experiments were conducted to evaluate the effectiveness of the proposed method. We trained our model on the COCO dataset and ensured that the experimental conditions were consistent. Exp. 1 represents the baseline, based on which we adopted the MLEM the transformer encoder based on the group axial attention mechanism (TEGA), and dynamic hyperparameter tuning training method based on Pareto efficiency (DHMP). A total of eight ablation experiments were performed, as shown in Table 2. Exp. 2 shows that the addition of MLEM resulted in a 1.6% increase in AP over DETR, demonstrating that multi-scale features have a more significant impact on the results. Exp. 3 shows that after adding TEGA, AP is improved by 0.9% compared to DETR, and compared to MLEM, the improvement is minimal, which indicates that the accuracy of the detection may depend on the pre-processing of the features; however, other experiments demonstrated that encoders have a material impact in terms of reducing model complexity and increasing inference speed. Exp. 3 shows that adding DHMP can make the model converge with a higher accuracy. A comparison of the results of Exps. 5–7 with those of Exp. 8, respectively, shows that each improvement is necessary to improve detection. Exp. 8 shows that applying all three improvements simultaneously significantly improves the accuracy, with a 2.4% improvement in AP.

**Table 2:** Ablation experiments number and results.

**ID**	**MLEM**	**TEGA**	**DHMP**	**AP**	**AP_50_**
Exp.1				0.519	0.721
Exp.2	✓			0.535	0.723
Exp.3		✓		0.528	0.725
Exp.4			✓	0.525	0.734
Exp.5		✓	✓	0.541	0.736
Exp.6	✓		✓	0.543	0.733
Exp.7	✓	✓		0.539	0.737
Exp.8	✓	✓	✓	0.552	0.741

As shown in Table 3, we conducted several groups of ablation experiments to demonstrate the effectiveness of Window G-Attention (WGA). Dataset 1 and Dataset 2 represent the COCO and PASCAL VOC datasets, respectively. Exp. 1 refers to the improved method without using WGA. By comparing the results of Exps. 1 and 3, as well as Exps. 2 and 3, we conclude that WGA contributes to improving FPS. It is worth noting that the degree of FPS improvement achieved by WGA varies across different datasets, which we believe is related to the distribution of objects within the images. In addition, we observed that the smaller the window size p of WGA, the lower the computational complexity and the higher the FPS, which is consistent with the conclusion we reached in the article.

**Table 3:** Ablation experiments number and results.

**ID**	**WGA**	**Dataset 1**	**Dataset 2**	**FPS (*p* = 4)**	**FPS (*p* = 8)**
Exp.1		✓		40	40
Exp.2			✓	41	41
Exp.3	✓	✓		46	44
Exp.4	✓		✓	45	43

**Figure 10:**
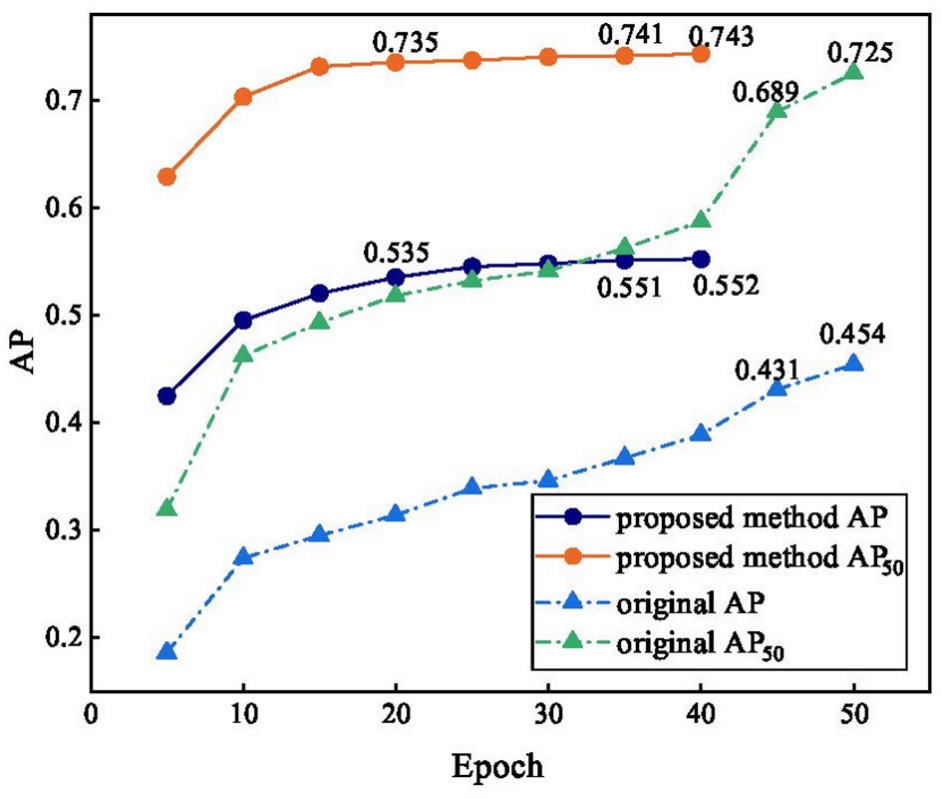
Comparison of accuracy trends before and after improvement.

Figure 10 reflects the accuracy trends before and after the improvement in the algorithm. The original method still has no convergence trend over 50 epochs, the AP and AP_50_ increase slowly, and the accuracy only reaches approximately 50% that of our method after 25 epochs, and the accuracy only reaches approximately 5% that of our method after 25 epochs. The accuracy of our method increased faster in the early stages of training, with AP and AP_50_ reaching 0.535 and 0.735, respectively, at epoch 20, which was higher than the accuracy of DETR at epoch 50. After 25 epochs, our method converges, indicating that DLMP is effective, and AP and AP_50_ finally reach 0.552 and 0.743, respectively.

### 3.4 Comparison with other methods

In this section, we compare our method with current mainstream algorithms on the COCO, PASCAL VOC and KITTI datasets, as shown in Tables 4–6. As shown in Table 4, the AP of our method is 0.552, and its AP_50_ is 0.741; both are the best, although AP_*S*_ is 1.4% lower than that of YOLOv8, and the actual epochs required for training are less than for YOLOv8. Compared with DN-Deformable-DETR and DINO-DETR, the proposed method maintains AP_*S*_ at the same level as the former and 0.4% higher than the latter while significantly reducing the GFLOPS and params. Compared to DETR, our method reduces the number of parameters by approximately 10% and improves the AP, AP_50_, and AP_*S*_ by 3.3%, 2.1%, and 4.5%, respectively, which is advantageous for DETR-like models.

**Table 4:** The results of comparison.

**Model**	**AP**	**AP_50_**	**AP_*S*_**	**Epoch**	**GFLOPs**	**Params**
YOLOv6 (Li et al., 2023)	0.542	0.727	0.327	100	150	59M
YOLOv7 (Wang et al., 2023)	0.546	0.733	0.331	100	104	36M
YOLOv8 (Talaat and ZainEldin, 2023)	0.551	0.739	**0.350**	100	165	43M
DETR (Carion et al., 2020)	0.519	0.720	0.291	110	187	41M
H-DETR (Zong et al., 2023)	0.539	0.732	0.332	70	268	42M
Anchor-DETR (Li et al., 2022)	0.521	0.721	0.294	80	172	39M
Deformable-DETR (Zhu et al., 2020)	0.539	0.726	0.316	50	173	40M
DAB-DETR (Liu et al., 2022)	0.543	0.732	0.325	50	256	44M
DN-Deformable-DETR (Li et al., 2022)	0.545	0.731	0.327	50	265	48M
DINO-DETR (Zhang et al., 2022)	0.550	0.737	0.332	**25**	279	47M
**Proposed method**	**0.552**	**0.741**	**0.336**	**40**	**161**	**37M**

Bold values indicate the best or second-best values.

Table 5 shows the experimental results of the different methods on PASCAL VOC. The AP of our method is 0.477, which is only lower than that of YOLOv8. The proposed method reaches convergence in 35 epochs, which is only higher than that of DINO-DETR, showing good convergence speed and more satisfactory detection accuracy.

**Table 5:** The results of comparison.

**Model**	**AP**	**AP_50_**	**AP_*S*_**	**Epoch**	**GFLOPs**	**Params**
YOLOv6 (Li et al., 2023)	0.571	0.672	0.316	100	150	59M
YOLOv7 (Wang et al., 2023)	0.473	0.676	0.319	100	104	36M
YOLOv8 (Talaat and ZainEldin, 2023)	0.477	0.678	**0.323**	100	165	43M
DETR (Carion et al., 2020)	0.433	0.643	0.251	110	187	41M
H-DETR (Zong et al., 2023)	0.443	0.653	0.268	70	268	42M
Anchor-DETR (Li et al., 2022)	0.438	0.652	0.256	80	172	39M
Deformable-DETR (Zhu et al., 2020)	0.461	0.663	0.273	50	173	40M
DAB-DETR (Liu et al., 2022)	0.465	0.669	0.278	50	256	44M
DN-Deformable-DETR (Li et al., 2022)	0.469	0.673	0.309	50	265	48M
DINO-DETR (Zhang et al., 2022)	0.475	0.678	0.318	**25**	279	47M
**Proposed method**	**0.478**	**0.687**	**0.321**	**35**	**161**	**37M**

Bold values indicate the best or second-best values.

Table 6 shows the results of different methods on the KITTI dataset. Our proposed method achieves the highest AP value of 0.556, representing a 3% improvement compared to the baseline method. Our method converges within 35 epochs, second only to DINO-DETR. In terms of AP_*S*_ evaluation, our method is only slightly behind YOLOv8 and is nearly on par with it, while having fewer model parameters and lower computational complexity.

**Table 6:** The results of comparison.

**Model**	**AP**	**AP_50_**	**AP_*S*_**	**Epoch**	**GFLOPs**	**Params**
YOLOv6 (Li et al., 2023)	0.546	0.745	0.347	100	150	59M
YOLOv7 (Wang et al., 2023)	0.548	0.766	0.352	100	104	36M
YOLOv8 (Talaat and ZainEldin, 2023)	0.554	0.821	**0.403**	100	165	43M
DETR (Carion et al., 2020)	0.526	0.722	0.286	110	187	41M
H-DETR (Zong et al., 2023)	0.539	0.732	0.332	70	268	42M
Anchor-DETR (Li et al., 2022)	0.537	0.729	0.327	80	172	39M
Deformable-DETR (Zhu et al., 2020)	0.541	0.736	0.349	50	173	40M
DAB-DETR (Liu et al., 2022)	0.545	0.739	0.353	50	256	44M
DN-Deformable-DETR (Li et al., 2022)	0.549	0.811	0.364	50	265	48M
DINO-DETR (Zhang et al., 2022)	0.553	0.819	0.381	**25**	279	47M
**Proposed method**	**0.556**	**0.823**	**0.397**	**35**	**161**	**37M**

Bold values indicate the best or second-best values.

**Figure 11:**
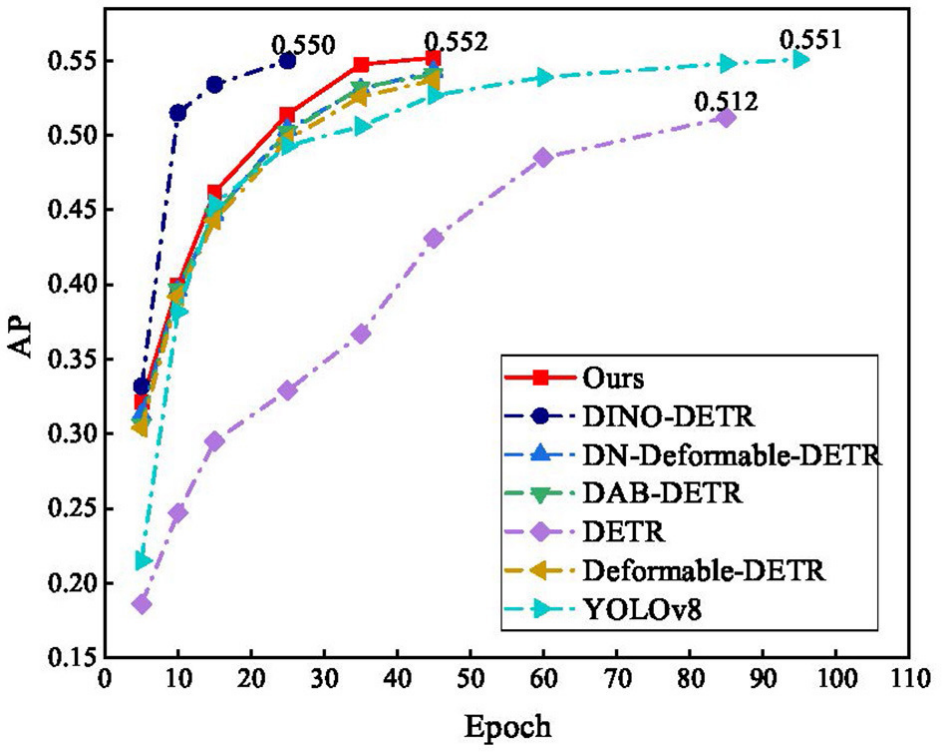
Results of comparison.

The convergence of the different algorithms during training is shown in Figure 11. The training of the proposed method essentially converged in fewer than 45 epochs, which is a remarkable improvement over DETR and YOLOv8. DINO-DETR converges in the 25th epoch, but in actual training, owing to its high complexity, the actual training of an epoch is approximately three times as long as that of the proposed method.

**Figure 12:**
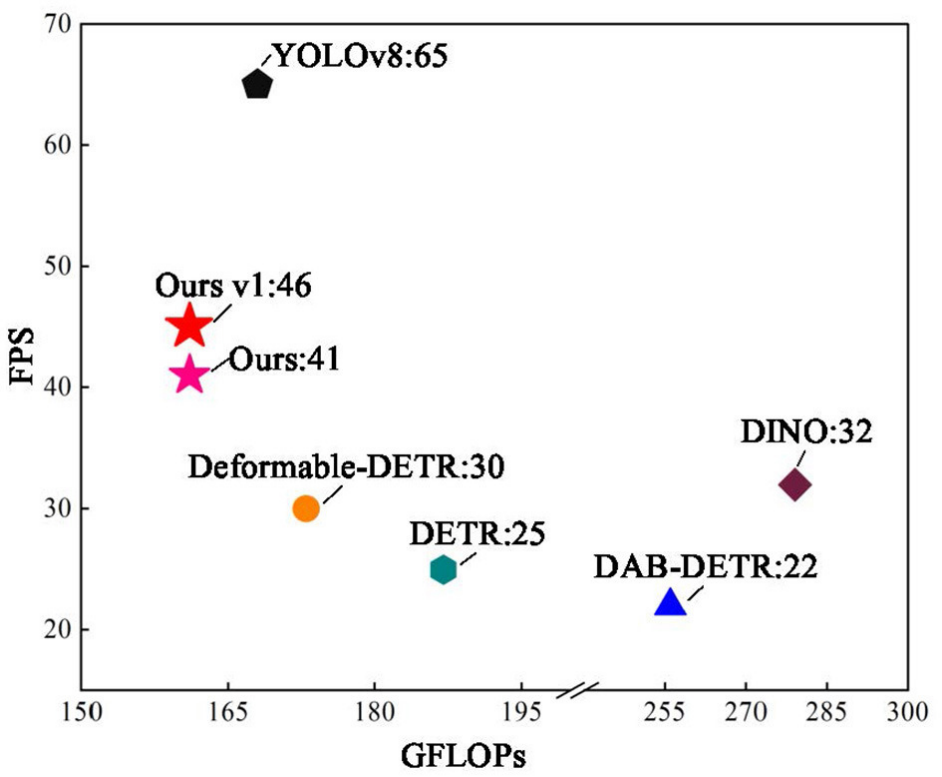
Comparison of FPS with different algorithms.

Figure 12 shows the relationship between the model computation GFLOPs and FPS and test images from the COCO and PASCAL VOC datasets, with an image size of 900 × 900. The proposed method had the smallest GFLOPs and best FPS in the DETR series. Version v1 uses Window G-Attention, and there is still a large gap in the FPS compared to YOLOv8; however, the FPS is improved by 84% compared to DETR.

In conclusion, the proposed method demonstrates robust performance across three distinct datasets. First, the AP values underscore the superior detection accuracy of the method, effectively identifying objects of varying scales in autonomous driving scenarios, including obstacles, vehicles, pedestrians, and traffic lights. Second, considering the inherent constraints of computational resources and energy consumption in autonomous driving hardware, our model is designed to minimize parameter size while achieving notable computational efficiency compared to mainstream object detection algorithms. With respect to the FPS metric, our method achieves the best performance among DETR-based algorithms, satisfying the stringent real-time detection requirements of autonomous driving systems. Furthermore, the proposed model is implemented using the PyTorch framework, ensuring seamless deployment on vehicle-embedded hardware. Notably, our method exhibits slightly lower performance on the AP_*S*_ metric compared to YOLOv8, likely due to the embedded data augmentation techniques employed by YOLOv8. This insight highlights a promising direction for further optimization and refinement of our algorithm.

### 3.5 Visualization

**Figure 13:**
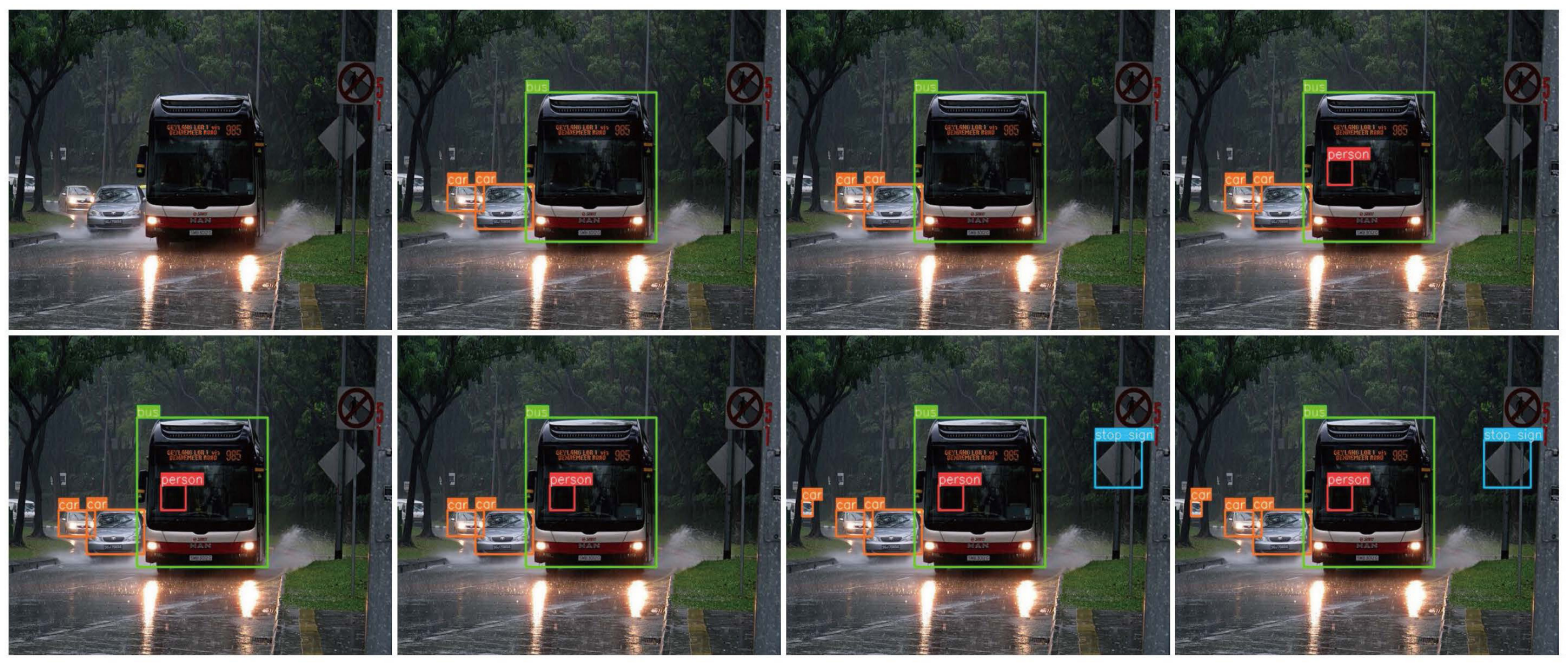
Visualization of the proposed method compared with current methods in suburban road scenes.

**Figure 14:**
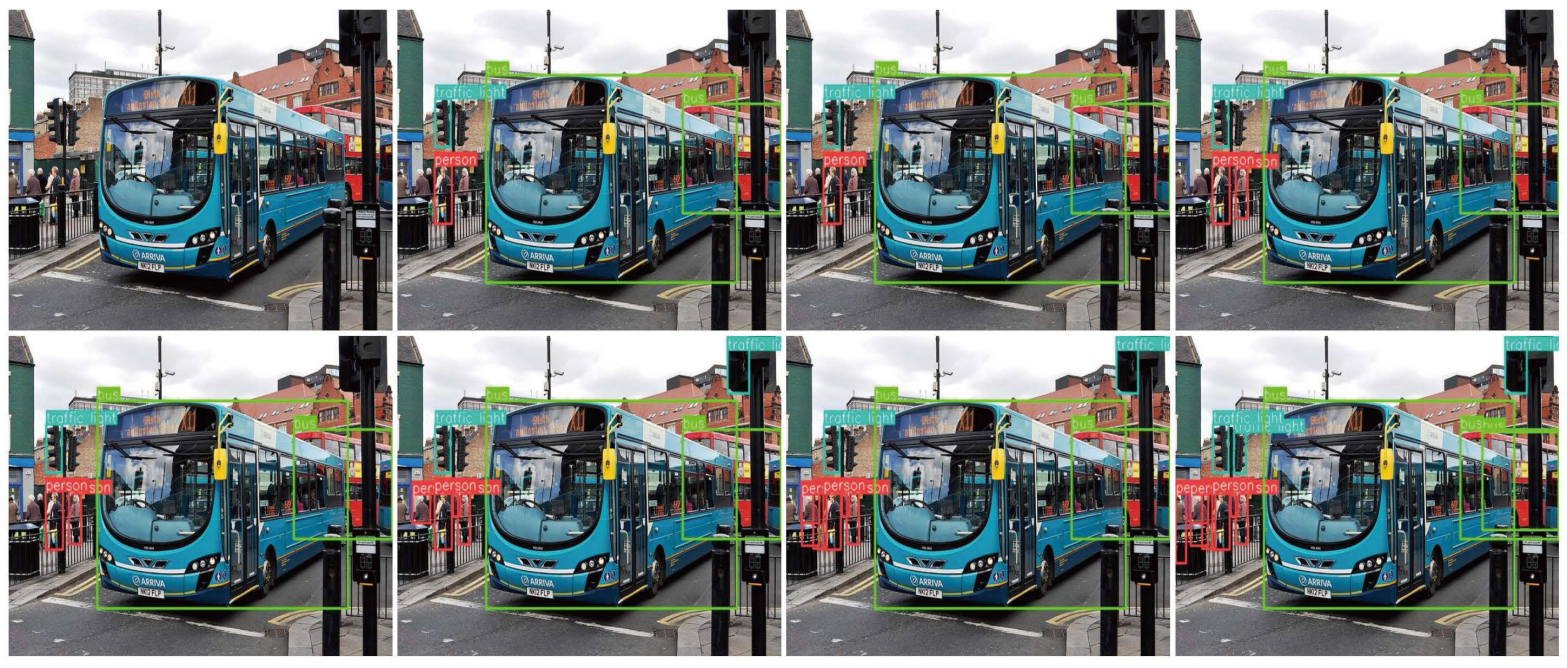
Visualization of the proposed method compared with current methods in suburban street scenes.

**Figure 15:**
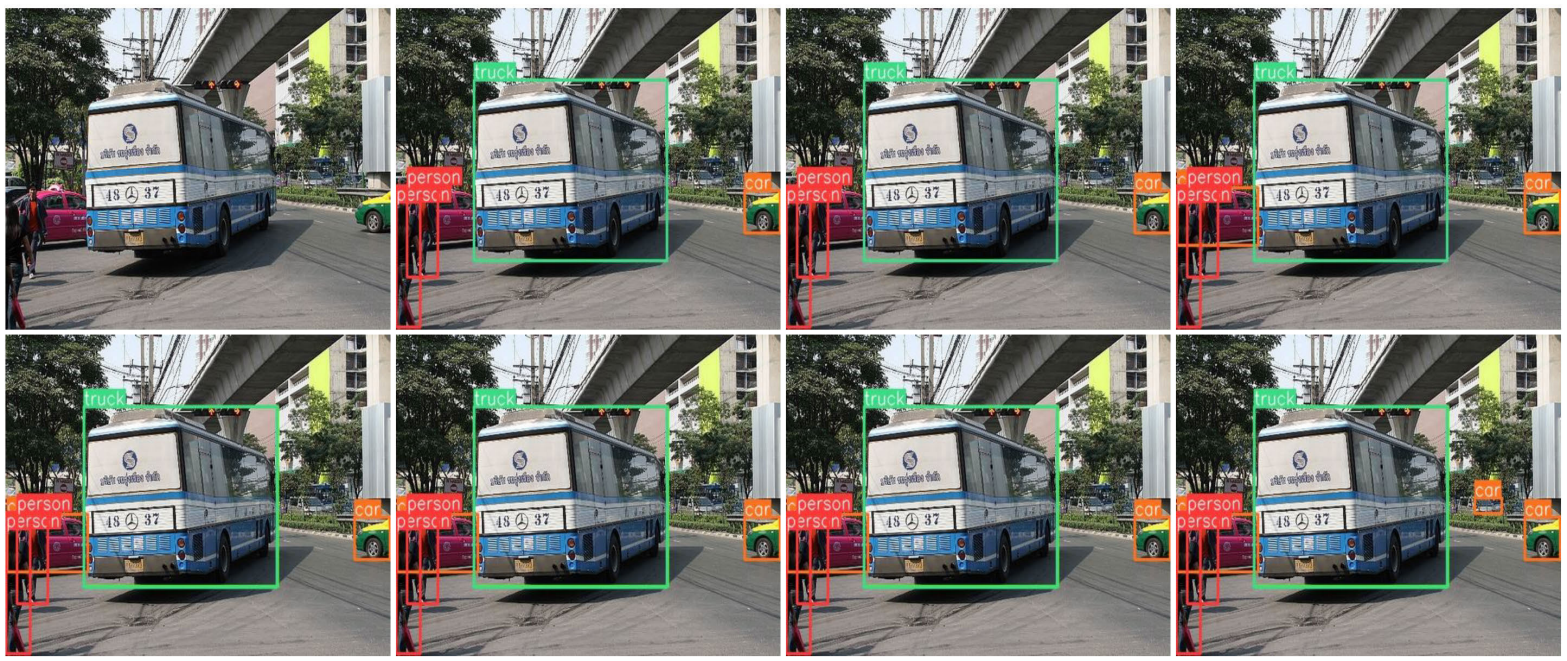
Visualization of the proposed method compared with current methods in suburban road scenes.

We tested the visualization of the different methods in various driving scenarios. The threshold for each detector was set to 0.6, and the performances are shown in Figures 13–15. Each image shows visualization of several approaches (Corresponding order from top to bottom, left to right): (a) the image to be detected, (b) DETR, (c) Deformable-DETR, (d) DAB-DETR, (e) DN-Deformable-DETR, (g) DINO-DETR (f) YOLOv8, and (h) the proposed method. According to the experimental results, the proposed method addresses the issues of omission and false detection caused by mutual occlusion, mutilation, and the small size of the target, and it has excellent adaptability to complex scenes.

Figure 13 shows a suburban road scene with clearer vehicle targets, but the problem of missing the car occurs in both (b) and (c), where the person in the car is a more difficult target to identify, and the remaining methods did not produce a missed detection. Both the proposed method and YOLOv8 detected traffic signs and the farthest vehicle.

Figure 14 shows a street scene, where people stand densely and the targets are small; however, the proposed method showed the least number of missed detections and best object detection. Figure 15 shows a city road scene, where there are cases of mutual occlusion and mutilation of detected objects, and only the proposed method successfully detected the vehicles behind the grass.

## 4 Conclusion

This paper presented an improved autonomous driving object detection method based on DETR. This approach includes a feature extraction technique that incorporates position-sensitive attention to improve multi-scale object detection. In addition, a transformer encoder with a group axial attention mechanism was developed to enhance the inference speed and reduce model computation. Furthermore, a dynamic hyperparameter tuning training method based on Pareto efficiency was implemented to adjust the training state of the loss function by dynamically modifying the weights. This approach aims to overcome the limitations associated with manually setting fixed weights, accelerate convergence, and improve model accuracy. Experimental results demonstrated that this approach outperforms traditional methods.

It should be emphasized that our method stands out for its exceptional inference speed compared to other DETR-like algorithms. However, it lags behind the YOLO series of algorithms in this regard. Achieving a high inference speed often results in a tradeoff with detection accuracy, posing a challenge for DETR-type algorithms to strike a balance between the two. In addition, our approach proved that the object detection model can be enhanced by synchronizing the training phases of the loss function. Developing a more intricate training strategy for the loss function is a promising prospect for future research. Finally, addressing faults in autonomous driving through fault detection, data reconstruction, decision optimization, and fault-tolerant mechanisms based on deep learning models is of great significance for improving the robustness and safety of autonomous driving systems.

## Data Availability

The raw data supporting the conclusions of this article will be made available by the authors, without undue reservation.
